# Muscle miRNAs are influenced by sex at baseline and in response to exercise

**DOI:** 10.1186/s12915-023-01755-3

**Published:** 2023-11-27

**Authors:** Danielle Hiam, Shanie Landen, Macsue Jacques, Sarah Voisin, Séverine Lamon, Nir Eynon

**Affiliations:** 1 Institute for Physical Activity and Nutrition, School of Exercise and Nutrition Sciences, Deakin University, Geelong, Australia; 2 Institute for Health and Sport (iHeS), Victoria University, Melbourne, Australia; 3 Hudson Institute of Medical Research, Melbourne, Australia; 4 Faculty of Health and Medical Sciences, Novo Nordisk Foundation Center for Basic Metabolic Research, University of Copenhagen, Copenhagen, Denmark; 5 Australian Regenerative Medicine Institute (ARMI), Faculty of Medicine, Nursing and Health Sciences, Monash University, Clayton, VIC 3800 Australia

**Keywords:** miRNA, Transcriptome, Sex differences, Skeletal muscle

## Abstract

**Background:**

Sex differences in microRNA (miRNA) expression profiles have been found across multiple tissues. Skeletal muscle is one of the most sex-biased tissues of the body. MiRNAs are necessary for development and have regulatory roles in determining skeletal muscle phenotype and have important roles in the response to exercise in muscle. Yet there is limited research into the role and regulation of miRNAs in the skeletal muscle at baseline and in response to exercise, a well-known modulator of miRNA expression. The aim of this study was to investigate the effect of sex on miRNA expression in the skeletal muscle at baseline and after an acute bout of high-intensity interval exercise. A total of 758 miRNAs were measured using Taqman®miRNA arrays in the skeletal muscle of 42 healthy participants from the Gene SMART study (23 males and 19 females of comparable fitness levels and aged 18–45 years), of which 308 were detected. MiRNAs that differed by sex at baseline and whose change in expression following high-intensity interval exercise differed between the sexes were identified using mixed linear models adjusted for BMI and W_peak_. We performed in silico analyses to identify the putative gene targets of the exercise-induced, sex-specific miRNAs and overrepresentation analyses to identify enriched biological pathways. We performed functional assays by overexpressing two sex-biased miRNAs in human primary muscle cells derived from male and female donors to understand their downstream effects on the transcriptome.

**Results:**

At baseline, 148 miRNAs were differentially expressed in the skeletal muscle between the sexes. Interaction analysis identified 111 miRNAs whose response to an acute bout of high-intensity interval exercise differed between the sexes. Sex-biased miRNA gene targets were enriched for muscle-related processes including proliferation and differentiation of muscle cells and numerous metabolic pathways, suggesting that miRNAs participate in programming sex differences in skeletal muscle function. Overexpression of sex-biased miRNA-30a and miRNA-30c resulted in profound changes in gene expression profiles that were specific to the sex of the cell donor in human primary skeletal muscle cells.

**Conclusions:**

We uncovered sex differences in the expression levels of muscle miRNAs at baseline and in response to acute high-intensity interval exercise. These miRNAs target regulatory pathways essential to skeletal muscle development and metabolism. Our findings highlight that miRNAs play an important role in programming sex differences in the skeletal muscle phenotype.

**Supplementary Information:**

The online version contains supplementary material available at 10.1186/s12915-023-01755-3.

## Background

Biological sex is a fundamental characteristic that has profound effects on physiological and molecular factors influencing nearly all complex traits [1, 2]. Sex differences arise from a combination of interacting factors such as differences in exposure to sex hormones, sex chromosome complement, epigenetic mechanisms and non-coding RNA programming. Whilst sex hormones and the sex chromosomes are the major drivers of sex differences, the potential of non-coding RNAs, specifically microRNAs (miRNAs), in programming sex differences in muscle and other tissues is starting to come into focus [3, 4].

MiRNAs are short (~ 20 nucleotides) single-stranded RNA molecules that primarily act by repressing a target mRNA molecule and reducing the translation and expression of the corresponding protein [5]. As regulators of gene and protein expression, they play a crucial role in a variety of biological processes, such as cell development, cell proliferation, differentiation, apoptosis and cellular signalling. Sex differences in miRNA expression profiles have been found across multiple tissue including pancreatic islets [6], peripheral blood, brain and mucosa tissues [3]. There is, however, limited research into whether there are sex differences in miRNA expression in the skeletal muscle and what upstream mechanisms may be mediating these differences. Yet, the skeletal muscle is one of the top sexually differentiated tissues as it has the second highest number of genes (up to 3000) that are differentially expressed between the sexes [7, 8]. This indicates a potential role of fine-tuning mechanisms, such as miRNAs, which could be crucial in programming sex differences in gene expression profiles and the resulting phenotype in muscle.

Males and females display distinct muscle phenotypes. On a functional level, females have lower muscle mass and strength compared to males [9, 10]. On the molecular level, there are intrinsic sex differences in fibre type proportions, satellite cell numbers and substrate metabolism [11]. These functional and molecular differences result in females being more susceptible to the metabolic and functional decline associated with both age-related muscle loss and prolonged periods of disuse due to sedentary lifestyle or muscle pathologies [9, 11]. Exercise is a potent skeletal muscle stimulus and is therefore one of the most effective lifestyle management therapies for preventing age-related muscle loss and metabolic conditions, such as type 2 diabetes mellitus (T2DM) and cardiovascular disease [12]. Males and females display differences in musculoskeletal, cardiovascular, and metabolic responses to the same exercise regime [13]. These differences have been partially attributed to differences in sex hormones [14, 15], transcription factors [16] and more recently DNA methylation [17, 18]. Exercise is well known to modulate microRNA expression [19], but there is limited literature investigating the potential differences between males and females in the skeletal muscle miRNA expression following an acute exercise bout. Therefore, the aim of this study was to investigate the influence of sex on miRNA expression in the skeletal muscle at baseline and after an acute bout of high-intensity interval exercise.

## Results

There were no significant differences in age or body mass index (BMI). Males had a higher aerobic capacity (VO_2peak_), lactate threshold (LT) and W_peak_ than females. As expected, males also had greater levels of both free and total testosterone than females, whilst females had greater SHBG and estradiol levels than males (Table 1).

**Table 1:** Participant characteristics stratified by sex

	**Female**	**Male**	***p***
*n*	20	24	
**Age (years)**	34.61 (7.37)	31.96 (8.14)	0.27
**BMI (kg.m**^**−2**^**)**	23.45 (2.90)	24.98 (3.42)	0.12
**W**_**peak**_** (W.kg**^**−1**^**)**	3.18 (0.71)	3.90 (0.88)	0.005
**VO**_**2**_** (mL.kg**^**−1**^** min**^**−1**^**)**	42.81 (7.08)	48.35 (8.04)	0.02
**LT (W.kg**^**−1**^**)**	2.14 (0.61)	2.71 (0.66)	0.006
**Circulating sex hormones**			
**TT**	0.84 (0.29)	19.61 (5.11)	< 0.001
**fT**	9.96 (3.92)	473.63 (140.71)	< 0.001
**SHBG**	73.11 (33.60)	34.87 (15.44)	< 0.001
**Estradiol**	227.55 (217.01)	107.73 (24.66)	0.012

Results are presented as mean (SD). *P* values < 0.05 are considered significant

*BMI* Body mass index, *W* Watts, *LT* Lactate threshold, *TT* Total testosterone, *fT* Free testosterone, *SHBG* Sex hormone binding globulin

### The expression levels of miRNAs at baseline are different between males and females

**Fig. 1:**
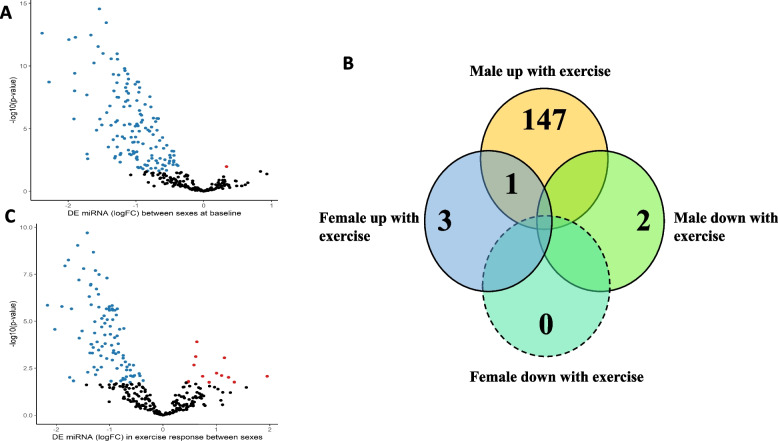
The skeletal muscle microRNA profile displays differences at baseline and a differential response to exercise between males and females. **A** Volcano plot: differences in microRNA expression levels between males compared to female at baseline. **B** Venn diagram: Overlap between miRNAs that were altered 3 h after an acute bout of exercise in females (female up-regulated miRNAs) and males (males up-regulated or down-regulated miRNAs). **C** Volcano plot: Interaction analysis revealed sex differences in miRNA response to an acute bout of exercise. Each point represents a microRNA. Red points indicate an increase in miRNA expression in males compared to females. Blue points indicate a decrease in miRNA expression in males compared to females. Black dots were not significant. Significance was set at FDR adjusted *p* value < 0.05. *N* = 44. Details of the specific miRNAs can be found in Additional file 1: Tables S1-5

At resting baseline (pre-exercise), the levels of 148 miRNAs were different between males and females in the skeletal muscle; 147 of these miRNAs displayed a lower expression in males compared with females and one miRNA displayed a higher expression in males compared to females (Fig. 1A).

### The response of miRNAs expression following exercise is different between males and females

The male and female cohort were first investigated separately to identify the miRNAs that were altered in response to high-intensity interval exercise in each sex. In the female cohort, four miRNAs were up-regulated, and no miRNAs were down-regulated in response to an acute bout of exercise (Fig. 1B, blue). In the male cohort, 148 miRNAs were up-regulated (Fig. 1B, yellow) and two miRNAs were down-regulated in response to an acute bout of exercise (Fig. 1B, green). One miRNA, miR-223-3p, was up-regulated in both males and females in response to exercise, whilst the remaining miRNAs were differentially altered specific to each sex in response to exercise. Secondly, we conducted interaction analysis to identify the miRNAs that were differentially induced in males vs females in response to exercise. We found a total of 111 miRNAs whose response to an acute bout of exercise significantly differed between the sexes (Fig. 1C).

### Sex-biased miRNAs target genes are enriched for pathways relating to muscle-related processes

**Fig. 2:**
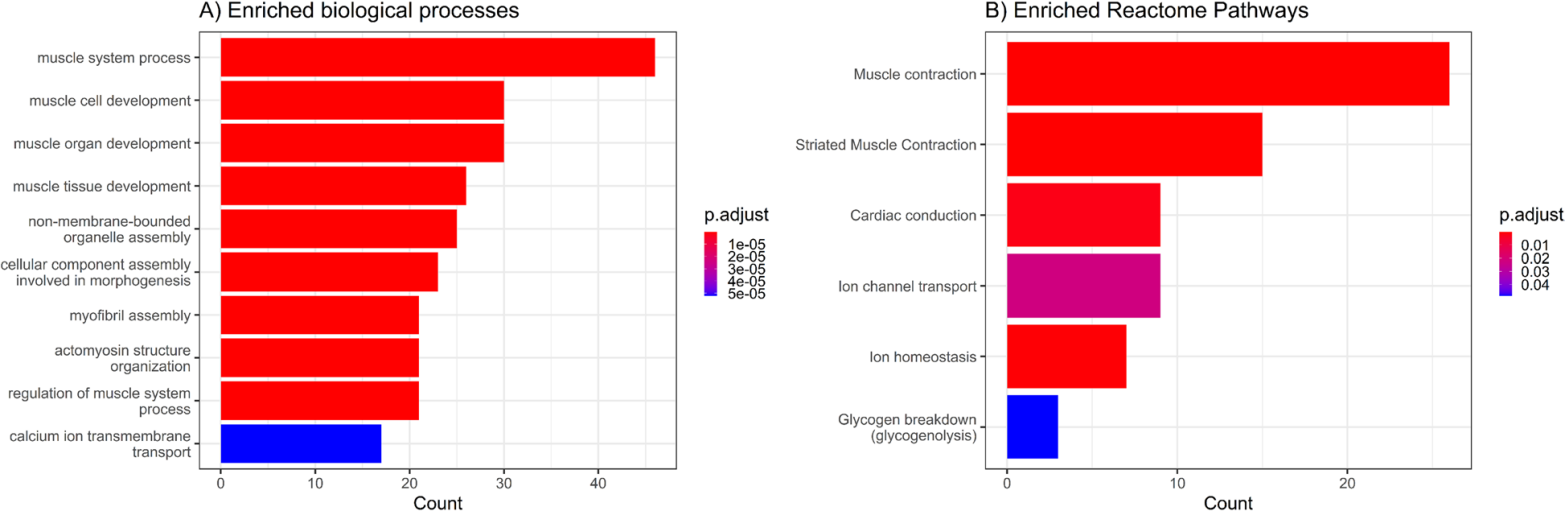
Gene set enrichment analysis of the skeletal muscle enriched gene targets of sex-biased miRNAs that differed in response to an exercise bout. **A** Top enriched gene ontology (GO) terms belonging to the ‘biological processes’ category. **B** Enriched Reactome pathways. Significance was set at FDR adjusted *p* value < 0.05. *N* = 44. Details of enriched pathways can be found in Additional file 1: Tables S7, S8

Overrepresentation analysis (ORA) was performed to identify the gene pathways targeted by the sex-biased miRNAs at baseline and those that differed in response to exercise between the sexes. This analysis was conducted on genes that were considered expressed in the skeletal muscle based on RNA sequencing data from the GTEx portal [20]. Overall, 123 Gene Ontology (GO) terms were overrepresented in the baseline analysis and 118 GO terms were overrepresented in the interaction analysis (Additional file 1: Tables S6, S7, S8). Similar pathways were targeted at both baseline and in response to exercise. Altogether, the GO pathways broadly belonged to the ‘biological processes’ categories including ‘muscle cell development’, ‘muscle contraction’, ‘muscle system processes’ and ‘muscle cell differentiation’ (Fig. 2A and Additional file 1: Tables S7, S8). In agreement with this, we found enrichment in the WikiPathway term ‘striated muscle contraction pathway’ and Reactome pathway terms ‘striated muscle contraction’, ‘ion homeostasis’ and ‘glycogen breakdown’ (*q* < 0.05) (Fig. 2B).

### Neither chromosomal location nor circulating sex hormones explained the basis of the sex-biased miRNAs expression in the skeletal muscle

We next investigated the possible basis of sex-biased miRNAs expression in the skeletal muscle. A Fisher test was performed to investigate whether sex-biased miRNA genes (baseline and interaction analysis) were more likely to be encoded on the sex chromosomes compared to the autosomes. There was no difference in likelihood of the sex-specific miRNA gene being located on the sex chromosomes or autosomes at baseline or after an acute bout of exercise (Additional file 2: Fig. S2). Sex hormones can regulate gene expression via signalling directly through sex hormone response elements located across the genome, and there are known hormone response elements in the promoters of some miRNAs [21, 22]. We therefore sought to understand whether circulating sex hormone concentration was associated with miRNAs expression levels detected in the muscle. There were no significant correlations between circulating estradiol or total testosterone levels and the expression of sex-specific miRNAs within each sex. Only one miRNA, hsa-miR-613, was negatively associated with free testosterone (fT) after FDR adjustment in males (Additional file 1: Tables S9).

### There was enrichment of specific transcription factors (TF) with binding sites that are located near the transcription start site (TSS) of sex-biased miRNAs

Transcription factors (TFs) have been shown to have sex-divergent targeting patterns across multiple tissues including the skeletal muscle [16]. We therefore sought to understand whether certain TF were more likely to regulate the sex-biased miRNAs in the muscle. For differentially expressed miRNAs at baseline, 23 TF binding sites were significantly enriched including TP53 (located near TSS of 35 sex-biased miRNAs), NFKB1 (located near TSS of 27 sex-biased miRNAs), NFE2L2 (located near TSS of 18 sex-biased miRNAs), SMAD3 (located near TSS of 17 sex-biased miRNAs) and TGFB1 (located near TSS of 14 sex-biased miRNAs) (Additional file 1: Tables S10). For sex-biased miRNAs in response to exercise, there was enrichment for 11 TF binding sites in the putative transcriptional regulatory region of the miRNAs. These included TP53 (located near TSS of 26 sex-biased miRNAs), NFKB1 (located near TSS of 23 sex-biased miRNAs), SMAD3 (located near TSS of 15 sex-biased miRNAs) and TGFB1 (located near TSS of 13 sex-biased miRNAs) (Additional file 1: Tables S11).

### Overexpression of selected sex-biased miRNAs in skeletal muscle primary cell lines

**Fig. 3:**
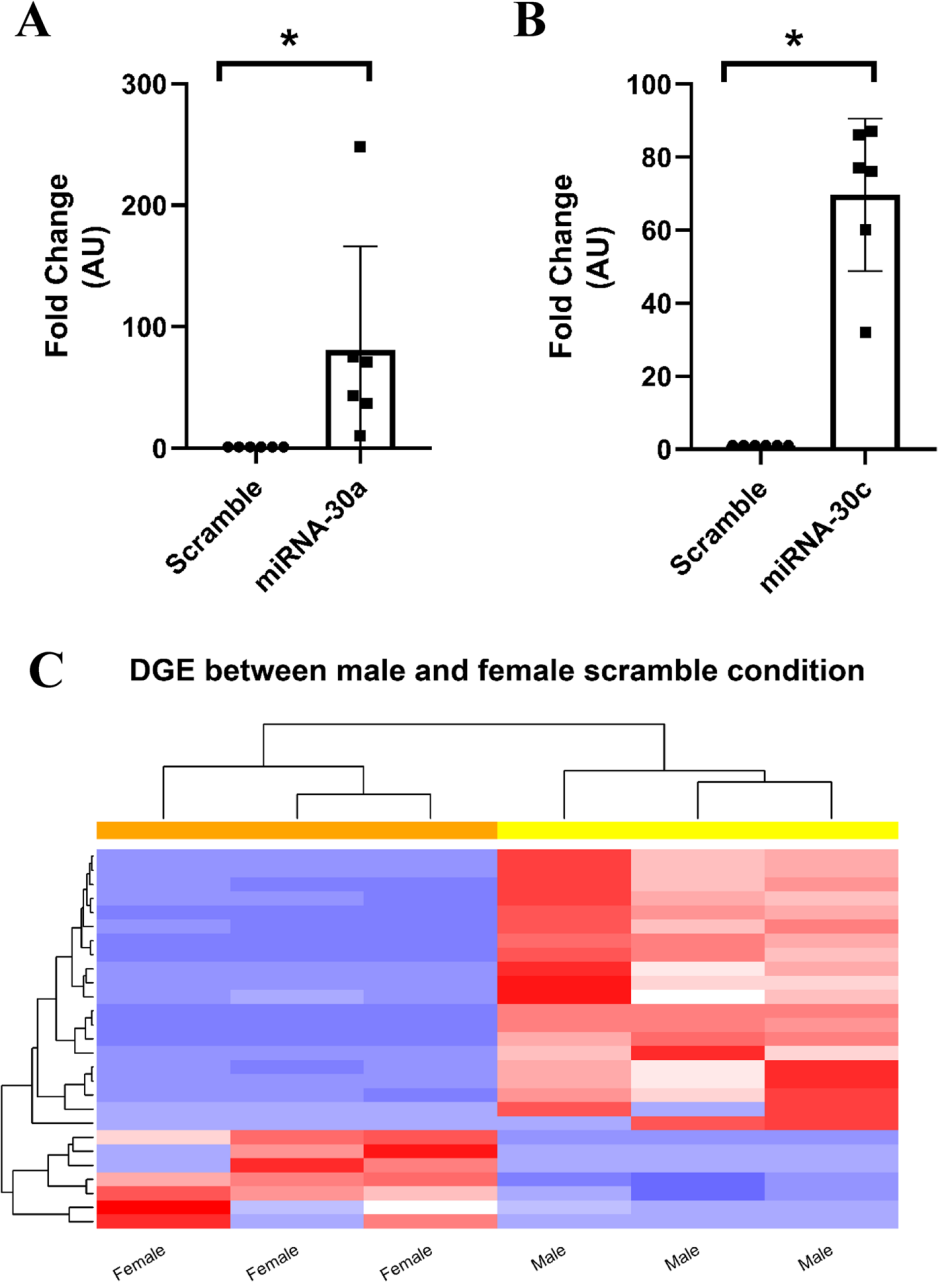
miRNA overexpression in skeletal muscle primary cells. QRT-PCR analysis of miR-30a/30c expression levels containing either **A** miR-30a or **B** miR-30c. Fold change normalised back to negative control. *P < 0.05. Data is expressed as mean ± SD**.** *N* = 6.** C** Heatmap of top 26 differentially expressed genes clustered by sex at baseline. Details of the specific genes can be found in Additional file 1: Table S1

All five miR-30 family members (miR-30a-5p, miR-30b-5p, miR-30c-5p and miR-30d-5p/-3p) displayed a sex-specific response to exercise (Additional file 1: Table S5). Two candidates in this family were selected, miR-30a-5p and miR-30c-5p, as they have yet to be studied in the skeletal muscle in the context of sex. Hsa-miRNA-30a-5p (miR-30a) and hsa-miRNA-30c-5p (miR-30c) were overexpressed in three female and three male primary skeletal muscle lines. Overexpression of miR-30a and miR-30c was confirmed by quantifying transcript levels using qPCR (Fig. 3A, B) and led to a 70-fold increase in miR-30a and 80-fold increase in miR-30c.We then conducted RNA sequencing to understand the transcriptome profile changes driven by these miRNAs and to explore the putative biological pathways.

### Overexpression of miRNA-30a resulted in differences in sex-specific gene responses

At baseline, and without miRNA overexpression, 26 genes were differentially expressed between primary muscle cells cultured from male and female donors (Fig. 3C).

**Fig. 4:**
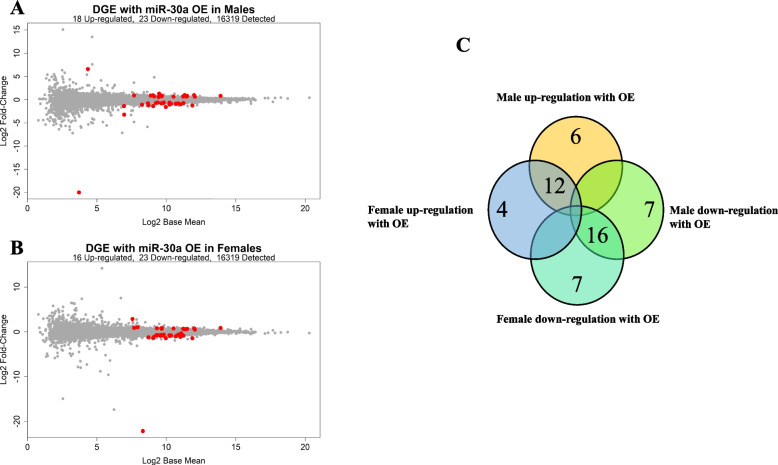
Differentially expressed genes with overexpression of miRNA-30a in human primary skeletal muscle. MA-plot of gene differentially expressed with overexpression of miR-30a in **A** males (*N* = 3) or **B** females (*N* = 3). **C** Venn diagram comparing the comparing DE genes with overexpression of miR-30a between the sexes. Significance at FDR adjusted *p* value set at *p* < 0.05. The complete list of genes can be found in Additional file 2: Tables S2-5

Overexpression of miR-30a resulted in differences in gene expression that were partly specific to the sex of the donor cells. In male-derived primary muscle cell lines, 18 genes were up-regulated and 23 genes were down-regulated with overexpression of miR-30a (Fig. 4A). In female-derived primary muscle cell lines, 16 genes were up-regulated and 23 were down-regulated with overexpression of miR-30a (Fig. 4B). Of these, 28 genes were similarly altered between male and female cell lines in response to overexpression of miR-30a and 24 genes were altered uniquely between male and female cell lines in response to overexpression of miR-30a (Fig. 4C, Additional file 2: Tables S2-5).

### Overexpression of miRNA-30c resulted in differences in sex-specific gene responses

**Fig. 5:**
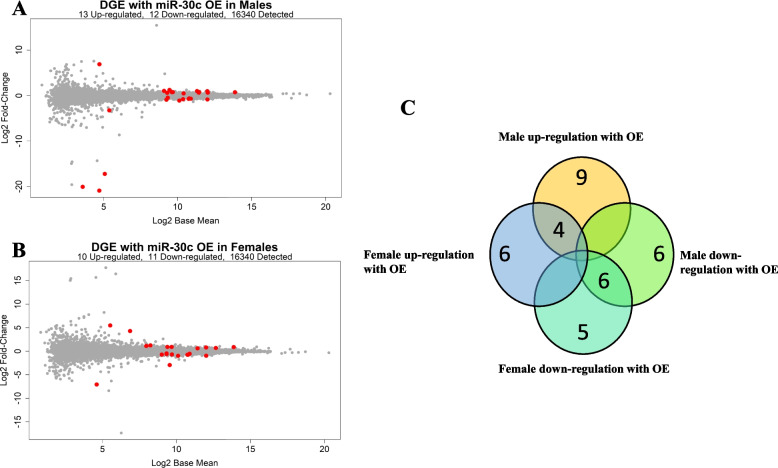
Differentially expressed genes with overexpression of miRNA-30c in human primary skeletal muscle. MA-plot of gene differentially expressed with overexpression of miR-30c in **A** males (*N* = 3) or **B** females (*N* = 3). **C** Venn Diagram comparing the comparing DE genes with overexpression of miR-30c between the sexes. Significance at FDR adjusted *p* value set at *p* < 0.05. The complete list of genes can be found in Additional file 2: Tables S6-9

Similar to miR-30a, overexpression of miR-30c resulted in differences in gene expression that were partly specific to the sex of the donor cells. In male-derived primary muscle cell lines, 13 genes were up-regulated and 12 genes were down-regulated with overexpression of miR-30c (Fig. 5A). In female-derived primary muscle cell lines, 10 were up-regulated and 11 were down-regulated with overexpression of miR-30c (Fig. 5B). Of these, 10 genes were similarly altered between male and female cell lines in response to overexpression of miR-30c (Fig. 5C). A total of 26 genes were altered uniquely between male and female cell lines in response to overexpression of miR-30c (Fig. 5C, Additional file 2: Table S6-7).

## Discussion

MiRNAs are necessary for skeletal muscle development [23] and have regulatory roles in determining skeletal muscle phenotype [24], which is one of the most sex-divergent tissues. Few studies have examined the effect of sex on the skeletal muscle miRNAome. Thus, in a screening approach, we conducted miRNA microarrays to profile the skeletal muscle miRNA signature across males and females at baseline and after an acute bout of high-intensity interval exercise. We found profound sex differences in the muscle miRNA profile, with an overwhelming number of miRNAs exhibiting a lower expression in males when compared to females. Three hours after an acute bout of high-intensity interval exercise, there were a greater number of differentially expressed miRNAs in males compared to females. Sex-biased miRNA gene targets were enriched for muscle-related processes such as myocyte proliferation and differentiation and metabolic pathways, indicating that miRNAs may play a role in programming the physiological sex differences in skeletal muscle pre- and post-exercise. We then investigated the potential upstream mechanisms, including chromosome location, sex hormones and transcription factors that may explain the basis of sex differences in the miRNAome. Neither chromosomal location of the miRNA gene nor circulating sex hormones explained sex differences in miRNAs expression in the skeletal muscle. The sex-biased miRNAs were enriched for 23 transcription factor (TFs) binding sites at baseline and 11 TFs after an acute bout of high-intensity interval exercise, indicating the potential role of specific TFs in regulating sex-biased miRNA expression. We further profiled two sex-biased miRNAs to elucidate the genes targeted by these miRNAs. Overexpression of miRNA-30a and miRNA-30c in human primary skeletal muscle cells resulted in changes to gene expression profiles that were partly specific to the sex of the cell donor. Altogether, this indicates that sex not only plays a role in regulating the expression of miRNAs but also in regulating their gene targeting patterns.

To our knowledge, this is the most extensive miRNA profiling analysis that has been completed in the skeletal muscle comparing males and females at rest or following an exercise intervention. Research into sex-specific differences in response to acute or chronic training adaptations is limited by the paucity of robust studies [13], especially in clinical settings. It is however established that some functional adaptations (VO2max, substrate metabolism) are sex-specific [13, 25]. In line with our findings, our group and others have also reported sex differences in proteome [26] and transcriptome [27], but not DNA methylome [26], in response to training. The response to acute and chronic exercise training is however complex, and males and females differ in some, but not all, aspects of physiological and molecular training adaptations. We reported 148 and 111 miRNAs displaying a sex bias at baseline and in response to an acute bout of high-intensity interval exercise, respectively. At baseline, an overwhelming majority of miRNAs were more highly expressed in females when compared to males including two myo-miRNAs (muscle-enriched miRNAs), miR-133b and miR-1-3p. This is in line with previous literature showing that expression levels of miR-133b were markedly higher in females compared to males at baseline [28]. Our findings also suggest that, after an acute bout of high-intensity interval exercise, males exhibited a greater change in the miRNA profile compared to females. In males, the myo-miRs-133a/b and 1-3p were up-regulated in response to acute exercise, which is in line with most [19, 29, 30], but not all studies [31]. The discrepancies between our data and Silver et al. [31] may be due to the differences in the intensity of the exercise bout between the studies (high-intensity interval exercise vs moderate intensity exercise), which can alter miRNA levels differentially [32]. In females, we observed no changes in the expression of miR-133a/b or miR-1-3p in response to acute exercise, in line with previous findings [31]. MiR-133a/b is under androgenic control in males but not females, which may explain these sex-specific responses [28]. While miR-133a/b did not associate with testosterone (free or total T) in the male cohort, it is also strongly modulated by aerobic fitness, which may override the regulatory effects of androgens in males [28]. According to Rapp et al. [33], our cohort was situated approximately in the 90th percentile for relative VO_2peak_ norms, indicating a high aerobic fitness, which may provide an explanation for this lack of association. Altogether, these data demonstrate the influence of sex on the miRNAome in muscle at both baseline and in response to acute exercise.

The sex chromosome complement (i.e. XX in biological females and XY in biological males) may contribute to sex differences in miRNA expression, as the X chromosome contains a higher density of miRNA genes compared to the Y chromosome [34, 35]. While many miRNAs genes are inactivated on the X chromosome, others are known to “escape” X inactivation and therefore would be expected to be more highly expressed in females compared to males [35–37]. Therefore, we investigated whether the sex-biased miRNA expression could be related to the genetic differences between the sexes. The current data did not indicate an overrepresentation of miRNAs genes on the X chromosome either at baseline or after high-intensity interval exercise which is consistent with findings in brain, colorectal tissue, blood and cord blood tissues [3]. These findings support the idea that sex chromosomes may not be the major contributor to the origin of sex-biased miRNA expression in the skeletal muscle. It should be noted that our findings were limited to miRNAs represented in the microarray array and therefore may not apply to the entire miRNAome of the skeletal muscle.

Sex steroid hormones can regulate miRNA expression directly via binding to their nuclear hormone receptors (AR, ERS1, ERS2) or indirectly by binding to the host gene promoters in intragenic miRNAs or through protein–protein interactions with other TFs [38]. There were no significant correlations between circulating estradiol or total testosterone levels and the expression of miRNAs within each sex. In line with previous literature, sex hormone receptors (AR, ERS1, ERS2) are not the sole regulators of sex-biased programming, which can also explain the lack of association [16]. Our putative analysis indeed revealed an enrichment of 23 TFs at baseline and 11 TFs in response to exercise that were not main sex hormone receptors. [16]. There was enrichment for TFs playing a role in determining the skeletal muscle phenotype. Specifically, TGFβ and its downstream mediator SMAD3 are involved in the skeletal muscle development [39, 40] and associated with altered substrate oxidation in the skeletal muscle [41–43]. TGFβ expression is influenced by sex in a range of tissues [44–46] and is known to regulate miRNA biogenesis [39]. At baseline, TGFβ putatively targeted 14 sex-biased miRNAs and 13 sex-biased miRNAs in response to a high-intensity interval exercise bout. One of these targets, miR-24, which displayed a sex bias at baseline and in response to exercise was repressed by TGFβ in C2C12 cells, leading to reduced expression of myogenic differentiation markers [47]. Further, TGFβ displays a sex-specific response to an acute bout of resistance exercise [48, 49]. Whether aerobic exercise elicits a similar sex-specific TGFβ response has not been investigated. Altogether, our results suggest that TFs that are not sex hormone receptors have sex-divergent targeting microRNA patterns in the skeletal muscle.

The miR-30 family plays a regulatory role in the differentiation of human skeletal muscle cells by targeting genes associated with cell cycle, proliferation and differentiation processes [50, 51]. We sought to investigate the effects of the miR-30 family on the myocyte transcriptome and to explore putative pathways in a sex-specific manner. We first wanted to establish whether the cell transcriptome exhibited sex differences in muscle primary cell lines. We therefore analysed the baseline differences in the transcriptome between male and female donor muscle cells and found that 26 genes were differentially expressed between the male and female donor cell lines. These included up-regulation of many Y-encoded (male-specific) genes that are considered to drive sex differences at a molecular level and included *Ddx3y* (DEAD box helicase 3), *Uty* (ubiquitously transcribed tetratricopeptide repeat gene on Y chromosome) and *KDM5D* (Lysine-specific demethylase 5D) amongst others [52, 53]. Interestingly, the X-encoded paralogs (*Ddx3x,* and *KDM5C)* were not different between the sexes, which may be due to X chromosome inactivation ensuring dosage compensation across the sexes [54]. The sex-biased genes were enriched for GO categories including immune system processes (antigen processing and presentation), gonad development (oocyte development and differentiation), DNA methylation and phenotypic switching which are known to display sex differences and sexual dimorphism (Additional file 2: Fig. S3). This finding adds to the existing evidence [55, 56] that sex differences originating from the sex chromosome complement may be conserved in primary cell lines [56]. However, the extent of the maintenance of sex differences mediated by lifelong exposure to different levels of gonadal sex hormones is still to be elucidated [52]. These data provide novel evidence that some sex differences in gene expression may be maintained in primary cell lines [57–62].

## Conclusion

In conclusion, there were sex differences in the expression profile of skeletal muscle miRNAs both at baseline and in response to high-intensity interval exercise. These sex-biased miRNAs had regulatory roles in the skeletal muscle development but also displayed sex differences in their gene targeting patterns. Our findings highlight the important role that miRNAs play in programming sex differences in the skeletal muscle phenotype.

## Methods

### Participants

Samples were from the Gene and Skeletal Muscle Adaptive Response to Training (Gene SMART) cohort [63]. The detailed methodology has been previously published [64–66]. Briefly, 24 healthy males (age = 32 ± 8.1 years old; BMI = 25.0 ± 3.4 kg/m2) and 20 healthy pre-menopausal females (age = 34.6 ± 7.4 years old; BMI = 23.4 ± 2.9 kg/m2) participated in the study. Females were not taking any hormonal contraceptives and were tested in the early follicular phase (2–7 days post menses). This study was approved by the Human Ethics Research Committee at Victoria University (HRE13-223 and HRE 21–122), and all participants provided written informed consent.

### Aerobic capacity (graded exercise test)

Aerobic capacity was assessed by a graded exercise test (GXT) performed on an electronically braked cycle-ergometer (Lode-Excalibur sport, Groningen, the Netherlands) to measure maximal oxygen uptake (V̇O_2peak_) and peak power output (W_peak_). The V̇O_2peak_ was determined using a calibrated Quark CPET metabolic system (COSMED, Rome, Italy). The GXT was performed at a minimum of 48 h before the acute exercise session and biopsy collection. It consisted of 4-min stages separated by 30-s rest periods until voluntary exhaustion with incremental increases in resistance at each stage. Capillary blood samples were collected at the end of each 4-min stage and immediately after exhaustion and were analysed by the YSI 2300 STAT Plus system (Ohio, USA) to establish lactate concentration. Lactate threshold (LT) was calculated by the modified DMAX method as previously described [64]. The GXT was performed in duplicate at both baseline and after the intervention and the average was calculated for all parameters between the two tests. In addition, at baseline, participants performed a familiarisation test of the GXT.

### Diet control (48 h prior to testing)

To standardise diet across the participants and minimise the effects of this confounding factor, each participant was provided with an individualised pre-packaged diet 48 h prior to providing the blood samples [67, 68]. The energy content of the provided meals was calculated using the Mifflin-St Jeor equation using the participant’s body mass, height and age [69]. The content of the diets was based on the current Australian National Health and Medical Research Council (NHMRC) guidelines. Participants were asked to abstain from caffeine and alcohol throughout the 48-h diet as well as food consumption 12 h prior to blood and muscle collection.

### Blood collection

Venous blood samples were collected at rest via venepuncture or cannulation in BD SST Vacutainers (Becton and Dickson Company, USA). They were left at room temperature (10 min) before being centrifuged at 3500 rpm for 10 min at 4 °C. Serum was collected and stored at − 80 °C.

### Muscle collection

Muscle biopsies were taken from the participants vastus lateralis muscle under local anaesthesia (1% xylocaine) using a Bergström needle modified to include suction [70] at baseline and 3 h after the acute exercise bout. Muscle was obtained from the same leg at both timepoints. The muscle was immediately frozen in liquid nitrogen and stored at − 80 °C for subsequent analyses. A small piece of the muscle (~ 10–15 mg) was placed in serum-free Ham’s F-10 medium (Life Technologies) for primary muscle cell culture.

### Hormone analysis

As the age of our cohort was 18–45 years, we measured the two major circulating sex hormones for this life stage, testosterone, and E2 at resting baseline [71, 72]. The assays were completed in the accredited NATA and Royal College of Pathologists of Australasia (RCPA) pathology laboratory at Monash Health, Clayton, Australia. Testosterone was measured using high performance liquid chromatography–mass spectrometry (HPLCMS/MS) method using a liquid sample extraction (AB Sciex Triple Quad 5500 LC/MS/MS system). Estradiol (E2) was measured using a competitive binding immunoenzymatic assay performed on a Beckman Coulter Unicel DXI 800 analyser (Beckman Coulter, Lane Cove, NSW).

### Acute HIIE bout

Male and female participants completed HIIE on an electronically braked cycle ergometer (Velotron, Racer Mate Inc, Seattle, USA). Participants completed approximately 5 min of warm up at an intensity of their own choosing [range 25–60 W] and then cycled for 6 × 2-min intervals and this was interspersed with 1-min recovery periods at a power of 60 W (work-to-rest ratio of 2:1). Intensity was individually determined based on baseline GXT results and calculated as power at lactate threshold (LT) + 40% of the difference between peak aerobic power (Wpeak) and power at LT.

### MicroRNA profiling of the skeletal muscle

RNA was extracted with Tri-Reagent Solution (Ambion) as per manufacturer’s protocol. RNA concentrations (243.3 ng/μl ± 81.7 ng/μl) and quality (A260/280 ratio = 1.97 ± 0.07) were determined by using the Nanodrop 1000 Spectrophotometer (Thermo Fisher Scientific). A total of 360 ng of RNA was reverse transcribed as using Taqman microRNA RT kit and Megaplex RT primers, Human Pool A and B v3.0 (Life Technologies, Australia) as per manufacturer’s protocol. MiRNA expression was measured using the TaqMan array, Human microRNA A and B (Life Technologies, Australia). Collectively, these cards allow for the accurate quantitation of 758 human miRNAs. The data was first normalised using global normalisation in cloud-based ThermoFisher software which applies a constant scaling factor to every miRNA, so they all have similar median intensity minimising batch effect. The following conditions were used to remove miRNAs that were not expressed in the skeletal muscle for analysis: miRNAs with an average Cq greater than 35 across all samples were excluded (*n* = 105), miRNAs where more than 20% of the samples (18/88) had a Cq of 35 were excluded (*n* = 345). The remaining 308 miRNAs were used in the analysis. The Cq values were then transformed into arbitrary units (AU) using the following equation: AU = 0.5^Ct^10^10^. The cut-off for the relevant level of expression of each miRNA was set at a mean (Ct) < 35, as recommended by the manufacturer. Out of the 758 miRNAs measured, 450 (58%) were not expressed in the skeletal muscle and were excluded, leaving 308 miRNAs for further analyses.

### Human primary cells

#### Experiment overview

Human primary cells were established from muscle biopsies of three females (age: 35.3 ± 8.6 years) and three males (32.3 ± 11.6 years) from the Gene SMART cohort (see Additional file 2: Table S10). A small piece of the muscle biopsy (please see above for detailed biopsy method) was collected. Satellite cells were isolated by dissociation with 0.05% trypsin/EDTA (Life Technologies). Cells were plated on a flask coated with extracellular matrix (ECM; Sigma-Aldrich, Castle Hill, Australia) and allowed to proliferate to 70% confluence before passaging. Myoblasts were maintained in proliferation media [Ham’s F-10 medium (Life Technologies) containing 20% FBS, 25 ng/mL fibroblast growth factor (bFGF; Promega), 1% penicillin streptomycin and 0.5% amphoteromycin (Life Technologies)] at 37 °C and 5% CO_2_. Cells were passaged by mechanical disturbance twice and then frozen down in proliferation media and 10% DMSO. Upon thawing, cells were passaged once before myoblasts were purified using CD56 + Microbeads (Miltenyi Biotec) to eliminate fibroblasts and other cell populations as per manufacturer instructions. At 70–80% confluence, cells were seeded in triplicate into 6-well plates and were allowed to differentiate by replacing proliferation media to differentiation media [DMEM (Life Technologies) containing 2% HS (New Zealand origin; Gibco, Life Technologies) and 1% penicillin/streptomycin. Medium was replaced every 48 h. On day 5 of differentiation, myotubes were transfected with either 20 nM of miR-30a or miR-30c mimics or 20 nM of a mimic scramble (*mir*Vana™ miRNA mimics, ThermoFisher) using Lipofectamine® RNAiMAX Transfection Reagent (Ambion, Life Technologies) diluted 16.7X in Opti-MEM I reduced serum medium (Life Technologies). This solution was added to differentiation medium at a 10 × dilution for 24 h. After 24 h transfection, cells were harvested for RNA [73].

#### RNA extraction and RNA sequencing

RNA was extracted from human primary cells using AllPrep RNA/RNA/miRNA universal kit (Qiagen) according to manufacturer instructions. Prior to adding the extraction buffer, the media was removed, and wells were washed with PBS to remove any floating dead cells. RNA quantity and quality was established using the Agilent Tape Station (Agilent); the average sample yield was 167 ng/μL ± 76.8 ng/μL and the RIN average was 9.6 ± 0.2. Overexpression was confirmed by quantifying transcript levels via qPCR using TaqMan Advanced miRNA probes and was normalised by loading a consistent amount of total RNA in the RT reaction. Each donor cell line was pooled into a single sample for library preparation. The RNAseq libraries were prepared using the Illumina TruSeq Stranded Total RNA with Ribo-Zero Gold protocol and sequenced with 150-bp paired-end reads on the Illumina Novaseq6000 (Macrogen Oceania Platform). Reads underwent quality check with FastQC (v0.11.9); Kallisto (v0.46.1) was used to map reads to the human reference genome (*HomoSapien GRCh38)* and to generate transcript counts. Differential gene analysis was conducted using the R package DeSeq2 [74] using the model: Genes ~ Sex * Condition. Pre-filtering was undertaken to remove genes with less than 10 reads per million (RPM) in less than two samples from further analysis [74].

#### Statistical analysis

All data were analysed using Rstudio 4.1.3 [75]. Missing data were imputed using the kNN function in the *VIM* package [76]. To identify differences in miRNA expression between sexes at baseline and after exercise, linear mixed models were used and implemented in the *limma* package in R [77]. Participant ID was used as a blocking variable to account for repeated measures using the DuplicateCorrelation function. The main sources of variability as determined by principal component analysis were included in the models (Additional file 2: Fig. S1). Sex and time were grouped to create means model of the form $\mathrm{miRNA }\sim 0+\mathrm{ Sex}.\mathrm{Timepoint}+\mathrm{BMI}+\mathrm{Wpeak}$ and a contrast matrix was constructed. Four contrasts were examined and included the difference between males and female at baseline, what miRNAs changed 3 h after an acute bout of exercise separately in each sex and finally to determine the differential response of miRNAs to an acute bout of exercise between sexes.

We then ran putative analysis to understand whether sex chromosomes or sex hormones were potential upstream regulators of miRNAs displaying sex differences. We tested associations between expression of miRNAs and hormone levels *within* each sex to assess whether hormone levels could explain some of the variability in miRNA expression. This analysis was performed in males and females separately, as they show very different ranges of hormones (thus avoiding collinearity). The model was of the form $\mathrm{miRNA }\sim \mathrm{ Hormone}+\left(1|\mathrm{ID}\right)$. A Fisher test was performed to investigate if sex-biased miRNA genes were more likely to be encoded on the sex chromosomes or the autosomes. Putative gene targets of the miRNAs were detected with the R package *multi-MiR* [78], using only gene targets from the experimentally validated databases (miRecords, miRTarBase and TarBase). Overrepresentation analysis (ORA) was used for gene target enrichment and implemented by the R package *clusterProfiler* [79]. The background gene list used for the ORA analysis comprised all experimentally validated targets of the miRNAs detected in the microarray. Tissue enrichment of the validated gene targets was conducted in the R package *TissueEnrich* [20]*.* Finally, transcription factor-microRNA interactions were explored using *TransmiR v2.0* using the default methods. The putative transcriptional regulatory region was considered 300 bp upstream and 100 bp downstream of each miR TSS or was based on published chip-seq data [80].

We considered significant miRNAs, pathways, tissues and transcription factors significant with an FDR adjusted *p* value < 0.05. The following packages were also used in our analysis: *lme4* [81], *lmerTest* [82] and *tidyverse* [83]. The full R code can be found at https://doi.org/10.5281/zenodo.8412095.

### Supplementary Information

**Additional file 1: Table S1. **The response of miRNAs expression following exercise in males, **Table S2.** The response of miRNAs expression following exercise in females, **Table S3.** Comparison of expression levels of miRNAs at baseline between males and females, **Table S4.** Comparison of expression levels of miRNAs post exercise bout between males and females, **Table S5.** Interaction analysis: miRNAs whose response to an acute bout of exercise differed between sexes, **Table S6.** miRNA gene targets and skeletal muscle enriched genes, **Table S7.** Over-representation analysis of sex-biased miRNA gene targets at baseline, **Table S8.** Over-representation analysis of sex-biased miRNA gene targets from interaction analysis, **Table S9.** Correlation analysis of circulating sex hormones and sex-biased miRNAs, **Table S10.** Transcription factor enrichment analysis of sex-biased miRNAs at baseline, **Table S11.** Transcription factor enrichment analysis of sex-biased miRNAs from interaction analysis.

**Additional file 2: Figure S1. **Principal component analysis: A) Scree Plot, B) Eigencorplot: **Figure S2.** The proportion of the sex-biased microRNAs genes expressed across the autosomes compared to the sex chromosomes at A) baseline B) after an acute bout of exercise. **Figure S3.** Gene set enrichment analysis: Top enriched GO terms of baseline male and female donor primary cells.

**Additional file 3: Table S1. **Comparison of male and female at baseline (scramble condition), **Table S2.** Comparison of male scramble vs overexpression of miR-30a, **Table S3.** Comparison of female scramble vs overexpression of miR-30a, **Table S4.** Comparison of male overexpression miR-30a and female overexpression miR30a, **Table S5.** Interaction Analysis of miR-30a, **Table S6.** Comparison of male scramble vs overexpression of miR-30c, **Table S7.** Comparison of female scramble vs overexpression of miR-30c, **Table S8.** Comparison of male overexpression miR-30c and female overexpression miR30c, **Table S9.** Interaction Analysis of 30c. **Table S10.** Donor Characteristics.

## Data Availability

All data generated or analysed during this study are included in this published article, its supplementary information files and publicly available repositories (GEO: GSE242861 and https://doi.org/10.5281/zenodo.8412095).

## Abbreviations

BMI: Body mass index
Ddx3y: DEAD box helicase 3
E2: Estradiol
fT: Free testosterone
GO: Gene Ontology
GXT: Graded exercise test
HIIE: High-intensity interval exercise
KDM5D: Lysine-specific demethylase 5D
LT: Lactate threshold
miRNA: MicroRNAs
myo-miRNAs: Muscle-enriched miRNAs
SHBG: Sex hormone binding globulin
T2DM: Type 2 diabetes mellitus
TFs: Transcription factors
TSS: Transcription start site
TT: Total testosterone
Uty: Ubiquitously transcribed tetratricopeptide repeat gene on Y chromosome
W: Watts
