# Supplementary material for: Muscle miRNAs are influenced by sex at baseline and in response to exercise
Source: BMC Biol. 2023 Nov 27;21:273. doi: 10.1186/s12915-023-01755-3 (PMC10683325; doi:10.1186/s12915-023-01755-3)
Supplement: Supplementary file 2 — Additional file 2: Figure S1. Principal component analysis: A) Scree Plot, B) Eigencorplot: Figure S2. The proportion of the sex-biased microRNAs genes expressed across the autosomes compared to the sex chromosomes at A) baseline B) after an acute bout of exercise. Figure S3. Gene set enrichment analysis: Top enriched GO terms of baseline male and female donor primary cells. [file 12915_2023_1755_MOESM2_ESM.pptx]

## Slide 1
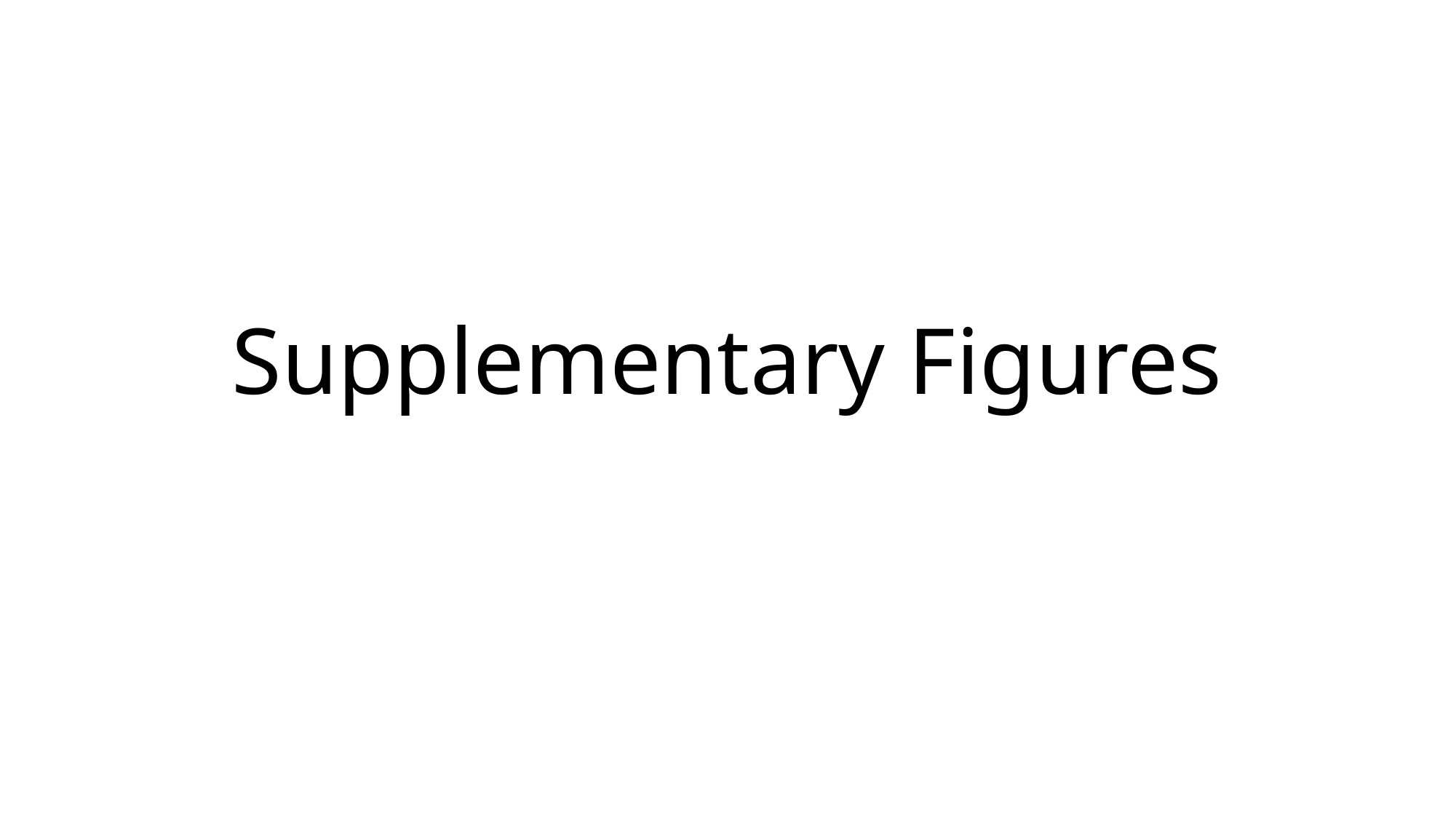

# Supplementary Figures

## Slide 2
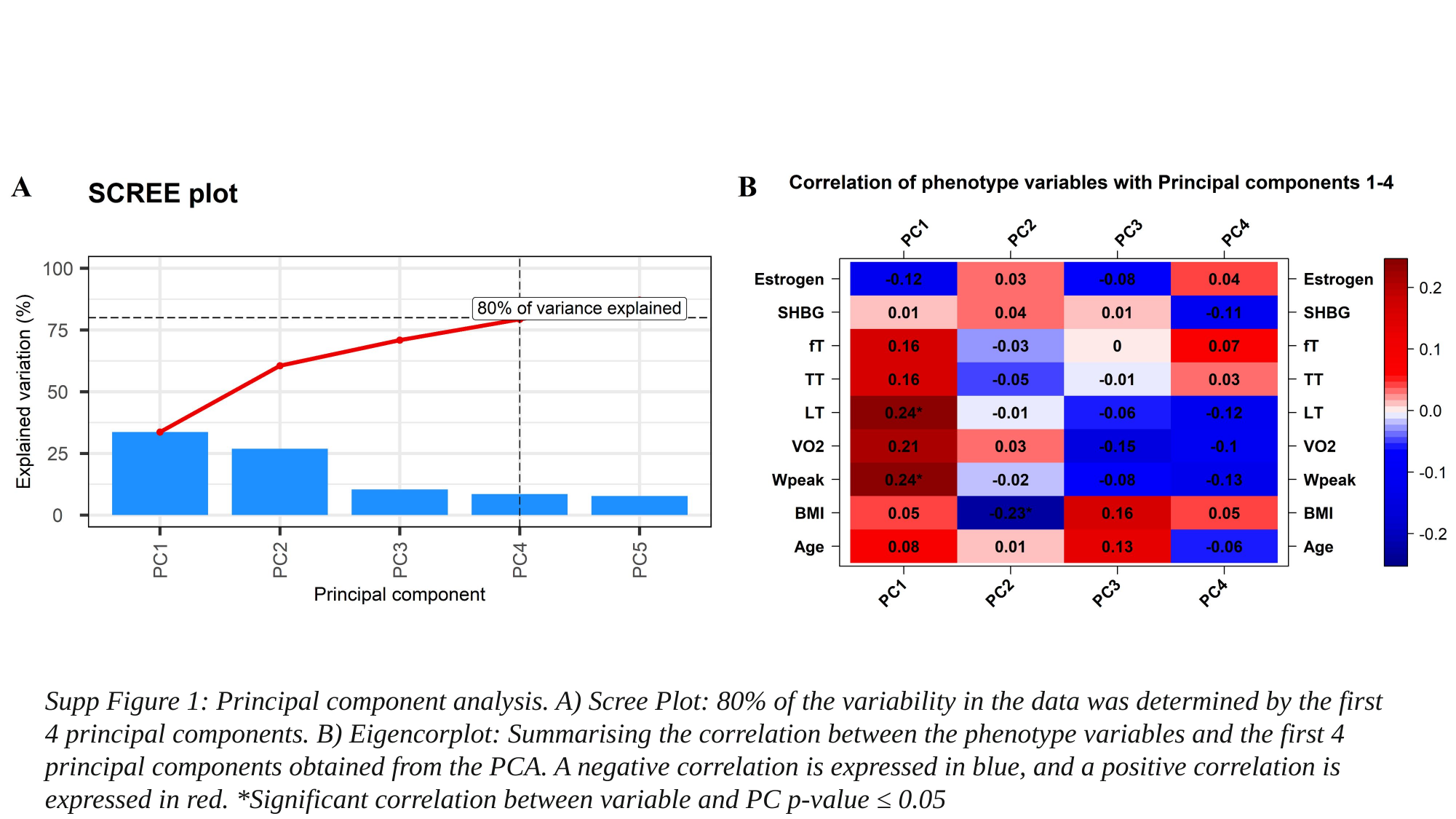

Supp Figure 1: Principal component analysis. A) Scree Plot: 80% of the variability in the data was determined by the first 4 principal components. B) Eigencorplot: Summarising the correlation between the phenotype variables and the first 4 principal components obtained from the PCA. A negative correlation is expressed in blue, and a positive correlation is expressed in red. *Significant correlation between variable and PC p-value ≤ 0.05

## Slide 3
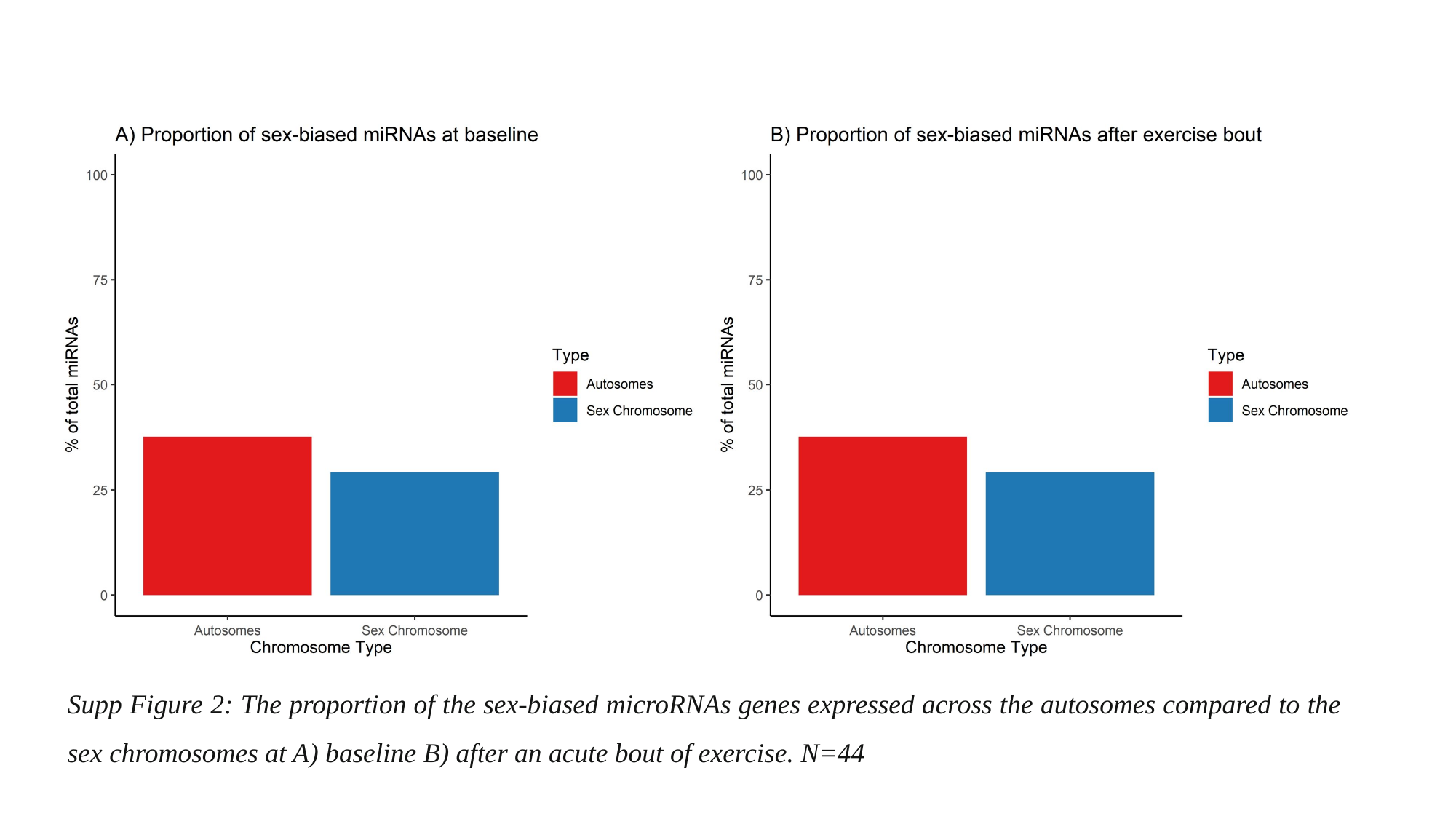

Supp Figure 2: The proportion of the sex-biased microRNAs genes expressed across the autosomes compared to the sex chromosomes at A) baseline B) after an acute bout of exercise. N=44

## Slide 4
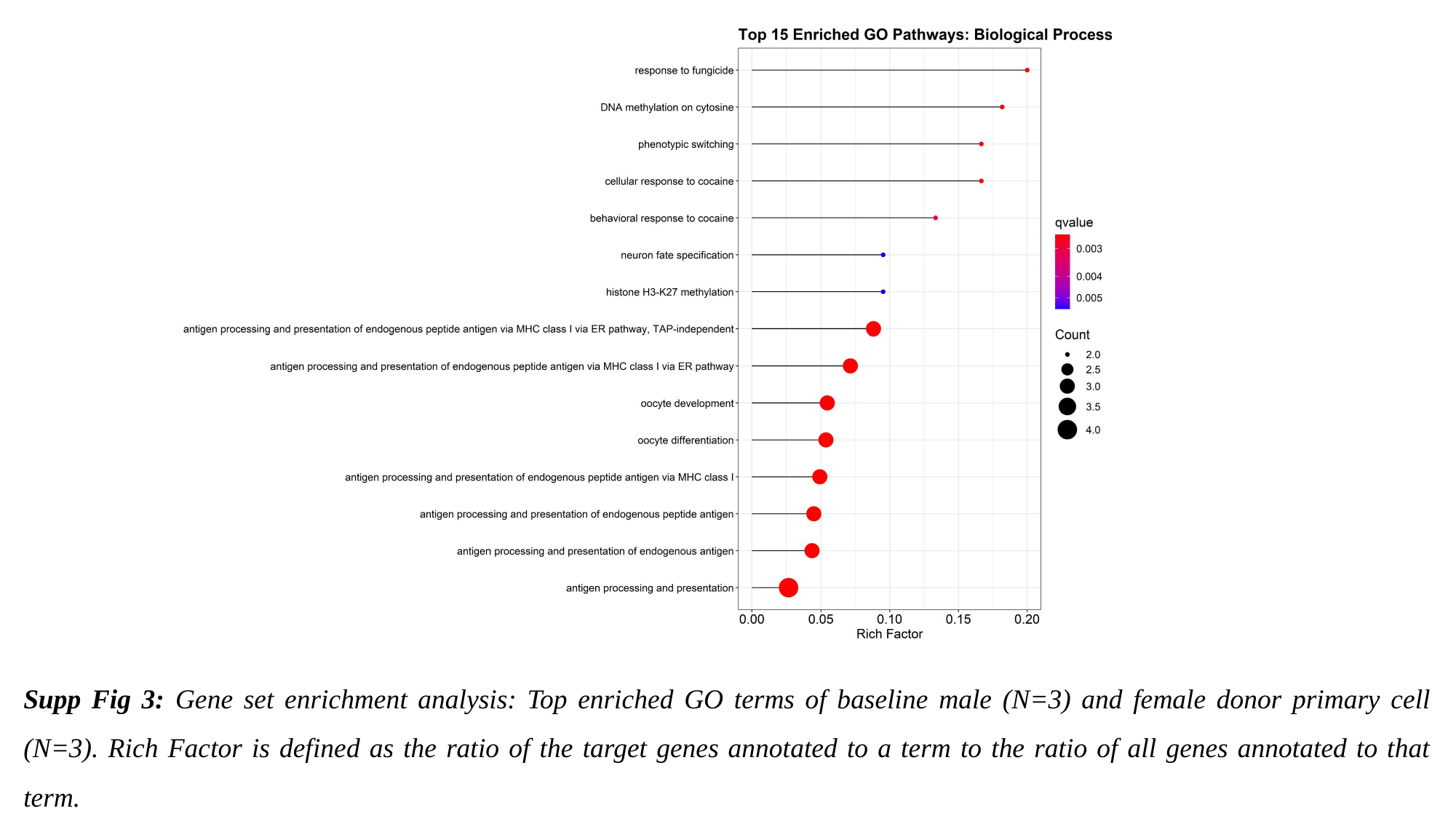

Supp Fig 3: Gene set enrichment analysis: Top enriched GO terms of baseline male (N=3) and female donor primary cell (N=3). Rich Factor is defined as the ratio of the target genes annotated to a term to the ratio of all genes annotated to that term.

## Slide 5
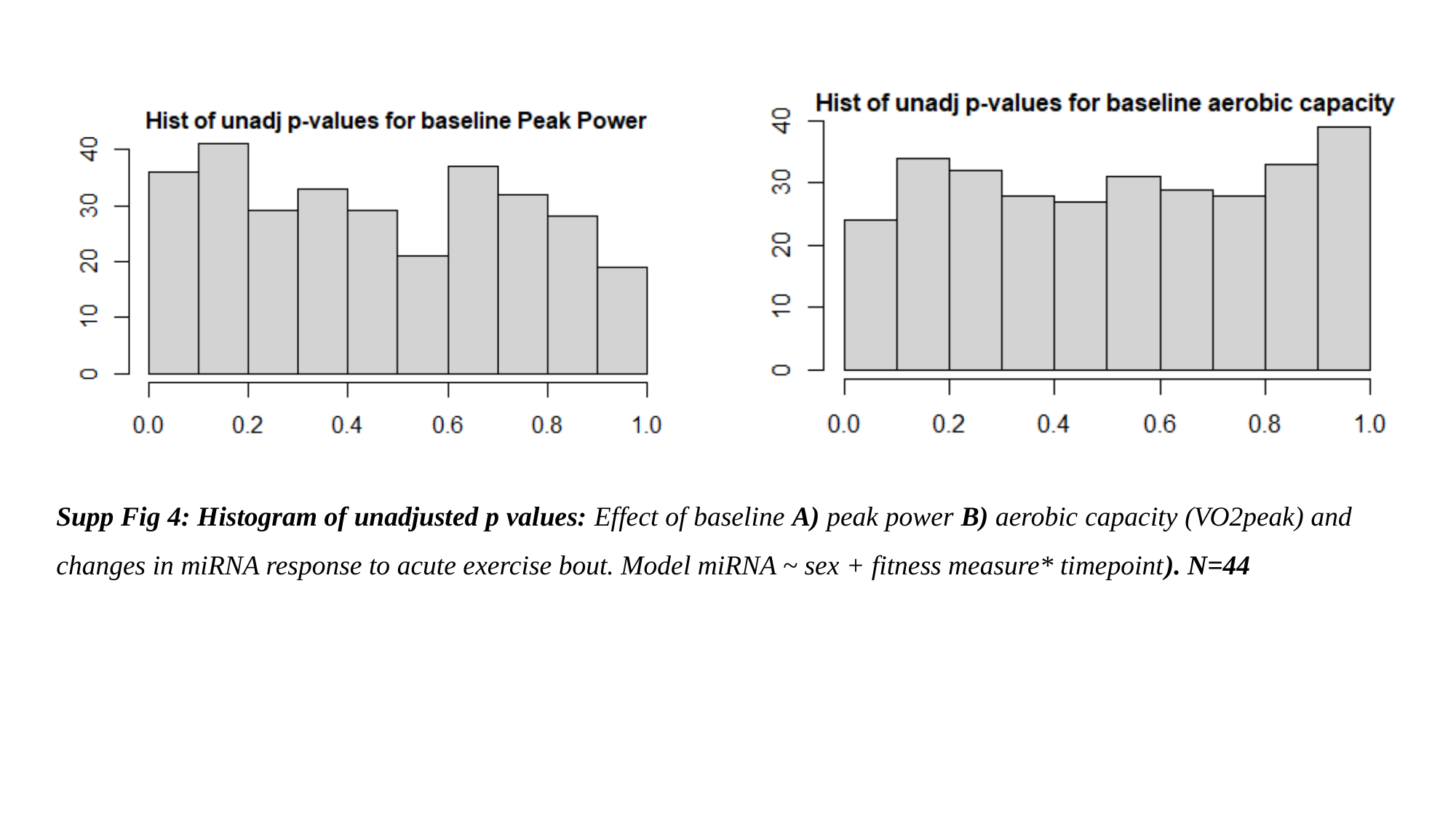

Supp Fig 4: Histogram of unadjusted p values: Effect of baseline A) peak power B) aerobic capacity (VO2peak) and changes in miRNA response to acute exercise bout. Model miRNA ~ sex + fitness measure* timepoint). N=44
